# Supplementary material for: Covariance regression with random forests
Source: BMC Bioinformatics. 2023 Jun 17;24:258. doi: 10.1186/s12859-023-05377-y (PMC10276920; doi:10.1186/s12859-023-05377-y)
Supplement: Supplementary file 1 — Additional file 1. Results of a simulation study comparing different ways of estimating the final covariance matrix [file 12859_2023_5377_MOESM1_ESM.pdf]

Additional file 1 for
Covariance regression with random forests

Cansu Alakus*, Denis Larocque, Aurélie Labbe

Final covariance matrix estimation

Random forests were introduced as a way to get predictions by averaging the predictions from many decision trees. In other words, random forest uses the in-bag training observations within a terminal node to get an estimate from that tree, and then uses the average of all trees' estimates as the final estimate for a new observation. Besides this traditional view, random forests can be seen as a way to find nearest neighbour observations that are close to the one we want to predict. For a new observation, the set of in-bag training observations that are in the same terminal nodes as the new observation forms the set of nearest neighbor observations, i.e. Bag of Observations for Prediction (BOP). We can define the BOP for a new observation $\mathbf{x}^*$ as

$$BOP(\mathbf{x}^*) = \bigcup_{b=1}^{B} I_b(\mathbf{x}^*),$$

where B is the number of trees and $I_b(\mathbf{x}^*)$ is the set of in-bag observations in the same terminal node as $\mathbf{x}^*$ in the bth tree.

In this paper, for a new observation $\mathbf{x}^*$, we form the set of nearest neighbour observations with the out-of-bag (OOB) observations. We can define the BOP_{oob} for a new observation as

$$BOP_{oob}(\mathbf{x}^*) = \bigcup_{b=1}^{B} O_b(\mathbf{x}^*),$$

where $O_b(\mathbf{x}^*)$ is the set of OOB observations in the same terminal node as $\mathbf{x}^*$ in the bth tree.

For a new observation, we can estimate the final covariance matrix using the alternative ways described above. We perform a simulation study with the four DGPs described in Data generating process subsection of the main paper to compare the performance of the four alternative ways of computing the final covariance matrix listed below.

1. Average of all trees' estimates computed with in-bag (IB) training observations
2. Average of all trees' estimates computed with out-of-bag (OOB) training observations
3. $BOP(\mathbf{x}^*)$ - BOP constructed with in-bag (IB) training observations
4. $BOP_{oob}(\mathbf{x}^*)$ - BOP constructed with out-of-bag (OOB) training observations

We can globally compare the accuracy over all scenarios with the percentage increase in MAE of a method with respect to the best method for a given run. For a given run, define MAE_i as the mean absolute error (MAE) of method i and MAE^* as the minimum MAE over the four alternative ways of estimating the final covariance matrix. The percentage increase in MAE for method i is computed as

$$100 \times \frac{MAE_i - MAE^*}{MAE^*}.$$

*Corresponding author. Department of Decision Sciences, HEC Montréal, 3000 chemin de la Côte-Sainte-Catherine, Montréal (Québec), Canada, H3T 2A7. E-mail: cansu.alakus@hec.ca

Smaller values for this measure indicate better performances. Supplementary Figure 1 presents the relative error of the alternative ways of computing final covariance matrix across 500 runs (5 n_{train} values $\times$ 100 replications) for each DGP. As demonstrated in Supplementary Figure 1, for DGP1, DGP2 and DGP3, $BOP_{oob}(\mathbf{x}^*)$ provides better accuracy compared to other three methods, whereas for DGP4, $BOP(\mathbf{x}^*)$ provides the best accuracy. In order to compare the results globally across all DGPs, Supplementary Figure 2 presents the relative error of the four alternative ways across 2,000 runs (4 DGPs $\times$ 5 n_{train} values $\times$ 100 replications). Globally, $BOP_{oob}(\mathbf{x}^*)$ provides slightly better accuracy among the the four alternative methods.

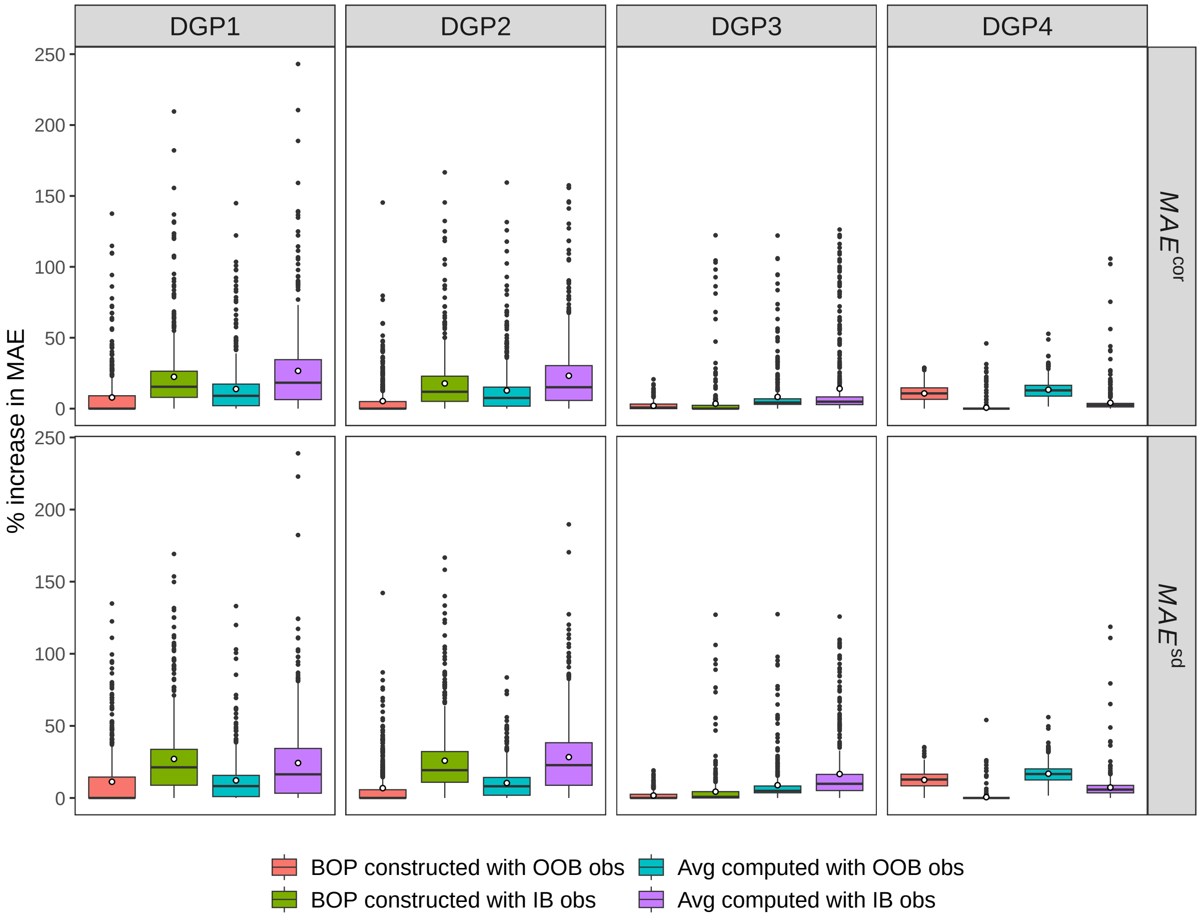

Supplementary Figure 1: Boxplots for the percentage increase in MAE of each alternative method compared to the minimum MAE for a given run across 500 runs for each DGP. The smallest is the percentage increase, the better is the method. Each white circle is the average of the relative MAE over 500 runs.

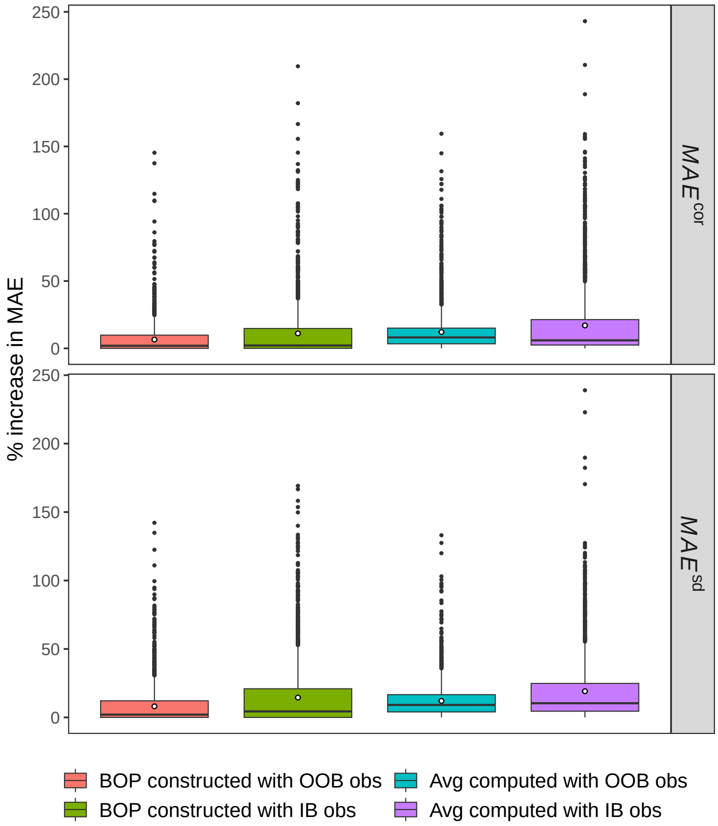

Supplementary Figure 2: Boxplots for the percentage increase in MAE of each alternative method compared to the minimum MAE for a given run across 2,000 runs for all DGPs. The smallest is the percentage increase, the better is the method. Each white circle is the average of the relative MAE over 2,000 runs.
