# Supplementary material for: Covariance regression with random forests
Source: BMC Bioinformatics. 2023 Jun 17;24:258. doi: 10.1186/s12859-023-05377-y (PMC10276920; doi:10.1186/s12859-023-05377-y)
Supplement: Supplementary file 2 — Additional file 2. Results of a nodesize tuning experiment [file 12859_2023_5377_MOESM2_ESM.pdf]

Additional file 2 for
Covariance regression with random forests

Cansu Alakus*, Denis Larocque, Aurélie Labbe

`nodesize` tuning

Supplementary figures 3 and 4 present the accuracy results for different levels of `nodesize` along with the proposed method which applies a `nodesize` tuning as described in the main paper. In the figures, the red boxplots illustrate the MAE results for the proposed `nodesize` tuning heuristic, and the remaining boxplots show the accuracy obtained when we set the `nodesize` to a specific value. For the set of `nodesize` levels to be searched in the proposed method, we have `nodesize`$= \{[(2^{-1}, 2^{-2}, 2^{-3}, \ldots)s] > q\}$ where q is the number of outcomes and s is the sub-sample size computed as $s = 0.632n_{train}$. As can be seen from the results in Supplementary Figure 3, as `nodesize` decreases, first MAE^{cor} and MAE^{sd} decrease and after a point increase for both DGP1 and DGP2. Since we have more levels of `nodesize` in the larger sample scenarios, it is easier to see this behaviour. For these two DGPs, smaller `nodesize` values do not mean better performance. As can be seen from Supplementary Figure 4, for DGP3 and DGP4, contrary to results of DGP1 and DGP2, MAE^{cor} and MAE^{sd} decrease as the `nodesize` increases. Hence, the best performing `nodesize` is mostly the smallest. Overall, when we compare the accuracy of the proposed `nodesize` tuning heuristic to the individual results of different `nodesize` values, we can see that it mostly performs well, especially for the larger sample sizes.

An example

As an example, we illustrate the steps of the `nodesize` tuning process for DGP2 with $n_{train} = 1000$. Let $s(1) < \ldots < s(M)$ be a set of increasing node sizes and are found as `nodesize`$= \{[(2^{-1}, 2^{-2}, 2^{-3}, \ldots)s] > q\}$ where $s = 0.632 * 1000 = 632$ and $q = 2$. Therefore, `nodesize`$= \{5, 10, 20, 40, 79, 158, 316\}$. After training separate random forests for this set of `nodesize` values (7 random forests), we compute the OOB covariance matrix estimates for each forest. Let $\hat{\mathbf{\Sigma}}^{s}_{\mathbf{x}_i}$ be the estimated covariance matrix for observation i when `nodesize`$= s$. Then, as described in the main paper, we compute

$$MAD_j = \frac{1}{632} \sum_{i=1}^{632} MAD\left(\hat{\mathbf{\Sigma}}^{s(j)}_{\mathbf{x}_i}, \hat{\mathbf{\Sigma}}^{s(j+1)}_{\mathbf{x}_i}\right), \quad j = \{1, 2, \ldots, 6\}.$$

In this example, MAD_j values are presented in Table 1. The smallest value is MAD_5 which is computed with $\hat{\mathbf{\Sigma}}^{s(5)}_{\mathbf{x}_i}$ and $\hat{\mathbf{\Sigma}}^{s(6)}_{\mathbf{x}_i}$. Therefore, the best `nodesize` is $s(5) = 79$.

Table 1: MAD_j values for the example. The smallest value is bold.

MAD_1	0.125
MAD_2	0.099
MAD_3	0.076
MAD_4	0.053
MAD_5	**0.048**
MAD_6	0.131

*Corresponding author. Department of Decision Sciences, HEC Montréal, 3000 chemin de la Côte-Sainte-Catherine, Montréal (Québec), Canada, H3T 2A7. E-mail: cansu.alakus@hec.ca

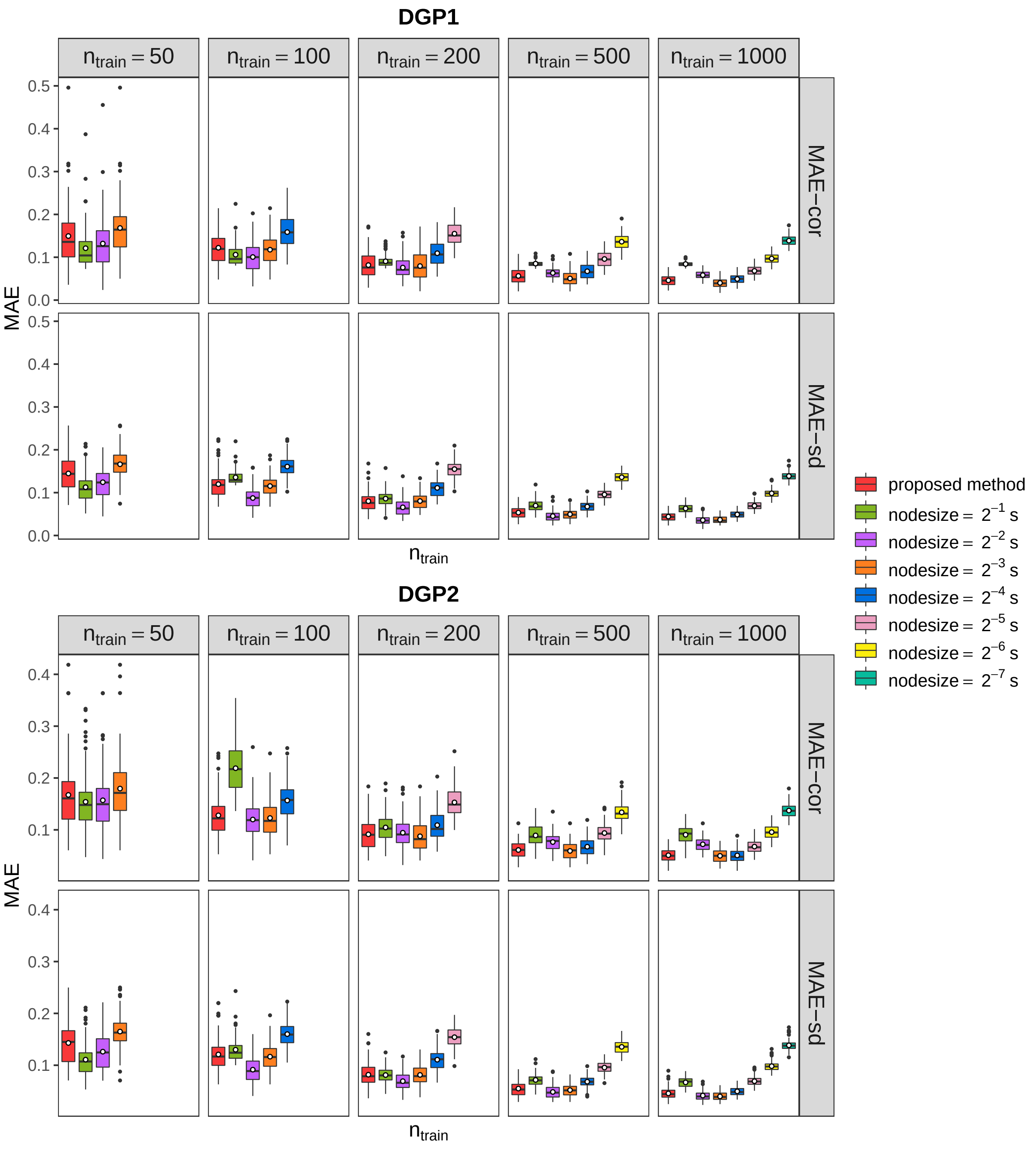

Supplementary Figure 3: MAE results for different `nodesize` values for DGP1 and DGP2. Smaller values of MAE^{cor} and MAE^{sd} are better. s is the sub-sample size, *i.e.* $s = .632 n_{train}$. Red boxplots illustrate the accuracy for the proposed `nodesize` tuning, and the rest of the boxplots show the accuracy obtained when we set the `nodesize` to a specific value.

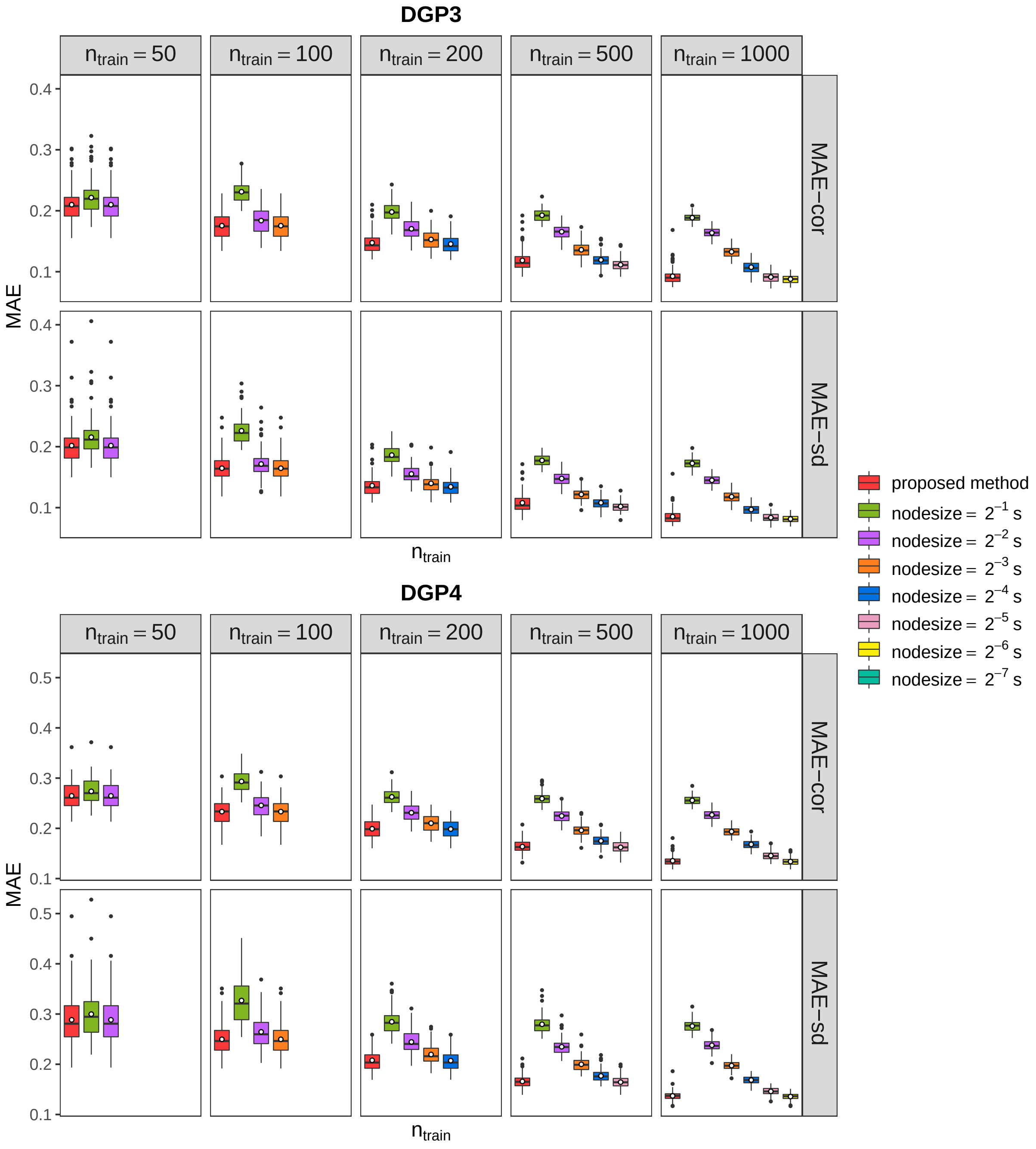

Supplementary Figure 4: MAE results for different `nodesize` values for DGP3 and DGP4. Smaller values of MAE^{cor} and MAE^{sd} are better. s is the sub-sample size, *i.e.* $s = .632n_{train}$. Red boxplots illustrate the accuracy for the proposed `nodesize` tuning, and the rest of the boxplots show the accuracy obtained when we set the `nodesize` to a specific value.
