# Supplementary material for: Covariance regression with random forests
Source: BMC Bioinformatics. 2023 Jun 17;24:258. doi: 10.1186/s12859-023-05377-y (PMC10276920; doi:10.1186/s12859-023-05377-y)
Supplement: Supplementary file 3 — Additional file 3. Details of the global significance test [file 12859_2023_5377_MOESM3_ESM.pdf]

Additional file 3 for
Covariance regression with random forests

Cansu Alakus*, Denis Larocque, Aurélie Labbe

Global significance test

The proposed global significance test is described in Supplementary Algorithm 1. After computing the unconditional and conditional covariance matrices, $\mathbf{\Sigma}_{root}$ and $\mathbf{\Sigma}_{\mathbf{x}_i}$, respectively, we compute the global test statistic with

$$T = \frac{1}{n}\sum_{i=1}^{n} d\big(\hat{\mathbf{\Sigma}}_{\mathbf{x}_i}, \mathbf{\Sigma}_{root}\big), \tag{1}$$

where $d(.,.)$ is computed as (2) in the main paper.

Supplementary Algorithm 1 Global permutation test for covariates' effects

1: Compute sample covariance matrix of $\mathbf{Y}$ in the root node, say $\mathbf{\Sigma}_{root}$
2: Train a RF with $\mathbf{X}$ and $\mathbf{Y}$
3: Estimate covariance matrices as described in Algorithm 1 of the main paper, say $\hat{\mathbf{\Sigma}}_{\mathbf{x}_i}$ $\forall i = \{1, \ldots, n\}$
4: Compute test statistic T as in (1)
5: **for** $r = 1 : R$ **do**
6: Permute rows of $\mathbf{X}$ to obtain $\mathbf{X}_r$
7: Train a RF with $\mathbf{X}_r$ and $\mathbf{Y}$
8: Estimate covariance matrices as described in Algorithm 1 of the main paper, say $\hat{\mathbf{\Sigma}}'_{\mathbf{x}_i}$ $\forall i = \{1, \ldots, n\}$
9: Compute test statistic with $T'_r = \frac{1}{n}\sum_{i=1}^{n} d\big(\hat{\mathbf{\Sigma}}'_{\mathbf{x}_i}, \mathbf{\Sigma}_{root}\big)$
10: **end for**
11: Approximate the permutation p-value with $p = \frac{1}{R}\sum_{r=1}^{R} I(T'_r > T)$
12: Reject the null hypothesis at level α when $p < \alpha$. Otherwise, do not reject the null hypothesis.

*Corresponding author. Department of Decision Sciences, HEC Montréal, 3000 chemin de la Côte-Sainte-Catherine, Montréal (Québec), Canada, H3T 2A7. E-mail: cansu.alakus@hec.ca
