# Supplementary material for: Covariance regression with random forests
Source: BMC Bioinformatics. 2023 Jun 17;24:258. doi: 10.1186/s12859-023-05377-y (PMC10276920; doi:10.1186/s12859-023-05377-y)
Supplement: Supplementary file 4 — Additional file 4. VIMP computation and figure presenting the performance of the estimated VIMP measures [file 12859_2023_5377_MOESM4_ESM.pdf]

Additional file 4 for
Covariance regression with random forests

Cansu Alakus*, Denis Larocque, Aurélie Labbe

Variable importance computation

Supplementary Algorithm 2 Variable importance computation

1: For original covariates $\mathbf{X}$ and responses $\mathbf{Y}$, estimate covariance matrices with the proposed method as described in Algorithm 1 in the main paper, say $\hat{\boldsymbol{\Sigma}}_{\mathbf{x}_i}$ $\forall i = \{1, \ldots, n\}$

2: Train a RF that uses a multivariate splitting rule based on the Mahalanobis distance with original covariates $\mathbf{X}$ to predict

$$\begin{bmatrix} \hat{\sigma}_{111} & \hat{\sigma}_{112} & \cdots & \hat{\sigma}_{1qq} \\ \hat{\sigma}_{211} & \hat{\sigma}_{212} & \cdots & \hat{\sigma}_{2qq} \\ \vdots & \vdots & \ddots & \vdots \\ \hat{\sigma}_{n11} & \hat{\sigma}_{n12} & \cdots & \hat{\sigma}_{nqq} \end{bmatrix}_{n \times \frac{q(q+1)}{2}}$$

where row i represents the upper triangular part of the estimated covariance matrix of observation i, $\hat{\sigma}_{ijk}$ represent the covariances in row j and column k of $\hat{\boldsymbol{\Sigma}}_{\mathbf{x}_i}$ $i = \{1, \ldots, n\}$, $j = \{1, \ldots, q\}$, $k = \{j, \ldots, q\}$.

3: Get the variable importance measures from this RF

Simulation results for variable importance

As stated in the main paper, Supplementary Figure 8 presents the average rank, from the estimated VIMP measures, for the important and noise variables groups for DGP3 and DGP4. The variable with the highest VIMP measure has rank 1. As rank increases, variable importance decreases. In all scenarios, the important variables have smaller average ranks than noise variables. As expected, the difference between the average ranks of important and noise variables increases with increasing sample size.

*Corresponding author. Department of Decision Sciences, HEC Montréal, 3000 chemin de la Côte-Sainte-Catherine, Montréal (Québec), Canada, H3T 2A7. E-mail: cansu.alakus@hec.ca

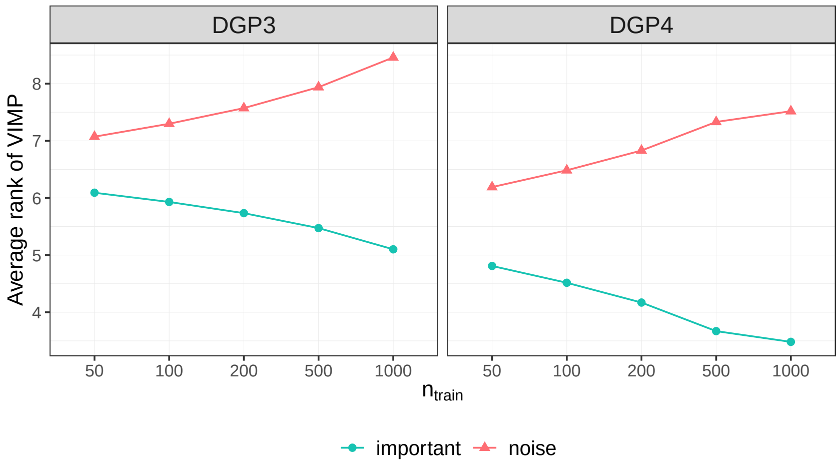

Supplementary Figure 8: Average ranks from estimated VIMP measures for DGP3 and DGP4. Smaller rank values indicate a more important variable (the most important variable has rank 1).
