# Supplementary material for: Covariance regression with random forests
Source: BMC Bioinformatics. 2023 Jun 17;24:258. doi: 10.1186/s12859-023-05377-y (PMC10276920; doi:10.1186/s12859-023-05377-y)
Supplement: Supplementary file 5 — Additional file 5. Details of the DGPs [file 12859_2023_5377_MOESM5_ESM.pdf]

Additional file 5 for
Covariance regression with random forests

Cansu Alakus*, Denis Larocque, Aurélie Labbe

Data generating process

In DGP1, the covariance matrix for the observation x_i is

$$\Sigma_{\mathbf{x_i}} = \mathbf{\Psi} + \mathbf{B}\mathbf{x}_i\mathbf{x}_i^T\mathbf{B}^T,$$

where $\mathbf{x}_i^T = (1, x_i)^T$, $\mathbf{B}_0 = [(1,-1)^T, (1,1)^T]$, $\mathbf{B} = \frac{w}{w+1}\mathbf{B}_0$, $\mathbf{\Psi}_0 = \mathbf{B}_0[(1,0)^T, (0,1/3)^T]\mathbf{B}_0^T$, $\mathbf{\Psi} = \frac{1}{w+1}\mathbf{\Psi}_0$ and $w = 1$.

In DGP3, the correlations are generated with all seven covariates according to a tree model with a depth of three and eight terminal nodes:

$$\begin{aligned}
\rho(\mathbf{x}_i) = {} & u_1 I\left(x_{i1} < 0, x_{i2} < 0, x_{i4} < 0\right) \\
& + u_2 I\left(x_{i1} < 0, x_{i2} < 0, x_{i4} \geq 0\right) \\
& + u_3 I\left(x_{i1} < 0, x_{i2} \geq 0, x_{i5} < 0\right) \\
& + u_4 I\left(x_{i1} < 0, x_{i2} \geq 0, x_{i5} \geq 0\right) \\
& + u_5 I\left(x_{i1} \geq 0, x_{i3} < 0, x_{i6} < 0\right) \\
& + u_6 I\left(x_{i1} \geq 0, x_{i3} < 0, x_{i6} \geq 0\right) \\
& + u_7 I\left(x_{i1} \geq 0, x_{i3} \geq 0, x_{i7} < 0\right) \\
& + u_8 I\left(x_{i1} \geq 0, x_{i3} \geq 0, x_{i7} \geq 0\right),
\end{aligned}$$

where the terminal node values are $u = (0.2, 0.3, 0.4, 0.5, 0.6, 0.7, 0.8, 0.9)$ and I is the indicator function. The variances are functions of ρ and computed as $Var(y_j|\mathbf{x}_i) = (1+\rho(\mathbf{x}_i))^j$, $j = \{1, \ldots, q\}$.

In DGP4, for an observation $\mathbf{x}_i$, we can generate the correlation with the logit model,

$$\rho(\mathbf{x}_i) = \frac{1}{1 + \exp\big(-(\beta_0 + \sum_{j=1}^{p} \beta_j x_{ij} + x_{i1}^2)\big)},$$

where β_0 is the intercept parameter fixed to $\beta_0 = -1$ and β_j are the weights for the covariates, fixed to $(1, 1-\frac{1}{p}, 1-\frac{2}{p}, \ldots, 1-\frac{(p-1)}{p})$. For an observation $\mathbf{x}_i$, the variance of each response is generated as $Var(y_j|\mathbf{x}_i) = (1+\rho(\mathbf{x}_i))^j$, $j = \{1, \ldots, q\}$.

*Corresponding author. Department of Decision Sciences, HEC Montréal, 3000 chemin de la Côte-Sainte-Catherine, Montréal (Québec), Canada, H3T 2A7. E-mail: cansu.alakus@hec.ca
