# Supplementary material for: Covariance regression with random forests
Source: BMC Bioinformatics. 2023 Jun 17;24:258. doi: 10.1186/s12859-023-05377-y (PMC10276920; doi:10.1186/s12859-023-05377-y)
Supplement: Supplementary file 6 — Additional file 6. Figures presenting the difference in MAE and accuracy evaluation with Stein’s loss [file 12859_2023_5377_MOESM6_ESM.pdf]

Additional file 6 for
Covariance regression with random forests

Cansu Alakus*, Denis Larocque, Aurélie Labbe

Difference in MAE between `CovRegRF` and competing methods

Supplementary figures 5 and 6 present the difference in MAE between *(red boxplots)* `covreg` and `CovRegRF`, and *(blue boxplots)* benchmark and `CovRegRF` results for 100 repetitions. In the boxplots, the values greater than 0 (above the dashed line) demonstrate that `CovRegRF` has smaller MAE than the competing method. On the contrary, the values less than 0 show that `CovRegRF` has larger MAE than the competing method.

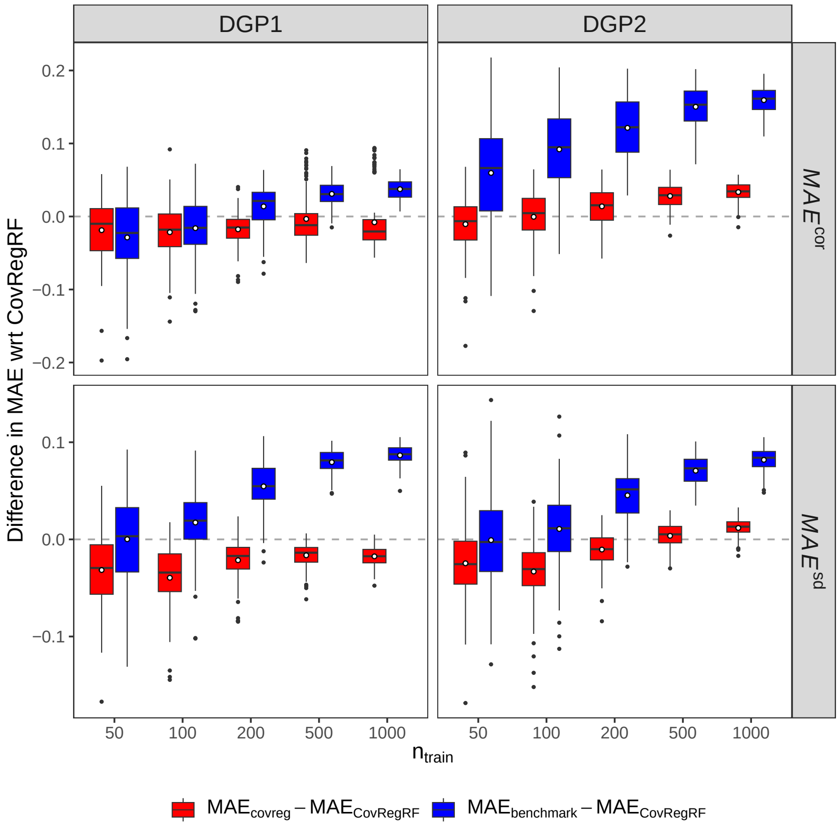

Supplementary Figure 5: Difference in MAE between *(red)* `covreg` and `CovRegRF`, and *(blue)* benchmark and `CovRegRF`. The dashed line is at 0.

*Corresponding author. Department of Decision Sciences, HEC Montréal, 3000 chemin de la Côte-Sainte-Catherine, Montréal (Québec), Canada, H3T 2A7. E-mail: cansu.alakus@hec.ca

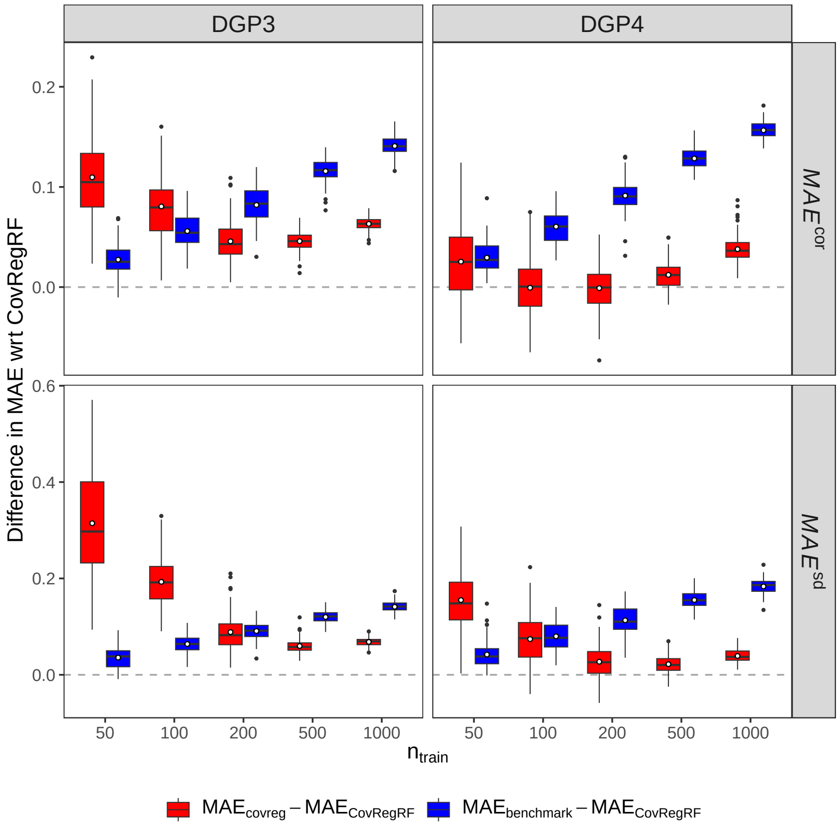

Supplementary Figure 6: Difference in MAE between *(red)* `covreg` and `CovRegRF`, and *(blue)* benchmark and `CovRegRF`. The dashed line is at 0.

Accuracy evaluation with Stein's loss

In addition to mean absolute errors (MAE) computed for the estimated correlations and standard deviations, we can compare each estimated covariance matrix $\hat{\mathbf{\Sigma}}_{\mathbf{x}_i}$ to its corresponding true matrix $\mathbf{\Sigma}_{\mathbf{x}_i}$ with Stein's loss which is the Kullback–Leibler divergence between two multivariate normal distributions with means zero and covariance matrices $\hat{\mathbf{\Sigma}}_{\mathbf{X}}$ and $\mathbf{\Sigma}_{\mathbf{X}}$,

$$l(\hat{\mathbf{\Sigma}}_{\mathbf{X}}, \mathbf{\Sigma}_{\mathbf{X}}) = \frac{1}{n_{test}} \sum_{i=1}^{n_{test}} \left(\mathrm{Tr}\left(\hat{\mathbf{\Sigma}}_{\mathbf{x}_i}^{-1} \mathbf{\Sigma}_{\mathbf{x}_i} \right) - \log\det\left(\hat{\mathbf{\Sigma}}_{\mathbf{x}_i}^{-1} \mathbf{\Sigma}_{\mathbf{x}_i} \right) - q \right)$$

where q is the number of responses. Each term in the sum becomes 0 when $\hat{\mathbf{\Sigma}}_{\mathbf{x}_i} = \mathbf{\Sigma}_{\mathbf{x}_i}$. Therefore, smaller Stein's loss values correspond to better covariance matrix estimates. Supplementary Figure 7 presents the Stein's loss for `CovRegRF`, `covreg` and benchmark for all DGPs for 100 repetitions.

For all DGPs, with increasing sample size, both the proposed method and `covreg` improve and the variance in Stein's loss decreases. For DGP1, `covreg` performs better compared to the proposed method for all sample sizes. However, the difference between `covreg` and the proposed method decreases with increasing sample size. For DGP2, for smaller sample sizes, `covreg` performs better whereas after $n_{train} = 500$, the proposed method performs slightly better. For DGP3, the proposed method performs significantly better than `covreg` for all sample sizes. Similarly, for DGP4, the proposed method has smaller Stein's loss compared to `covreg` for all sample sizes.

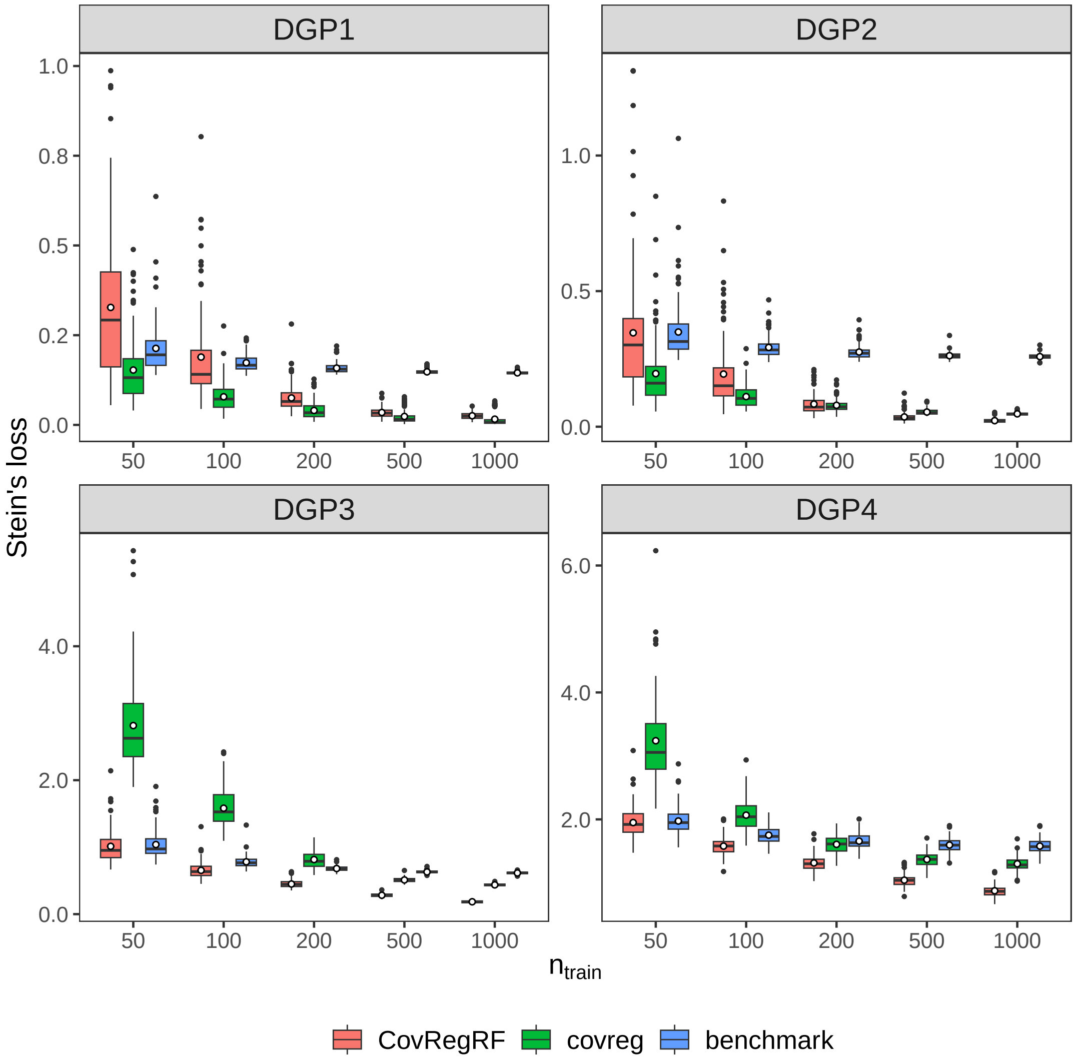

Supplementary Figure 7: Accuracy evaluation for four DGPs with Stein's loss. Smaller values of this metric are better.
