# Supplementary material for: Covariance regression with random forests
Source: BMC Bioinformatics. 2023 Jun 17;24:258. doi: 10.1186/s12859-023-05377-y (PMC10276920; doi:10.1186/s12859-023-05377-y)
Supplement: Supplementary file 7 — Additional file 7. Figure presenting the estimated variances of four thyroid-related hormones [file 12859_2023_5377_MOESM7_ESM.pdf]

Additional file 7 for
Covariance regression with random forests

Cansu Alakus*, Denis Larocque, Aurélie Labbe

Real data example

Supplementary Figure 9 presents the estimated variances of the four hormones (TSH, T3, TT4 and FTI) as a function of age, sex, and diagnosis. As we can see from the plots, the variances differ with diagnosis and sex, whereas age does not seem to have much effect on the estimated variances.

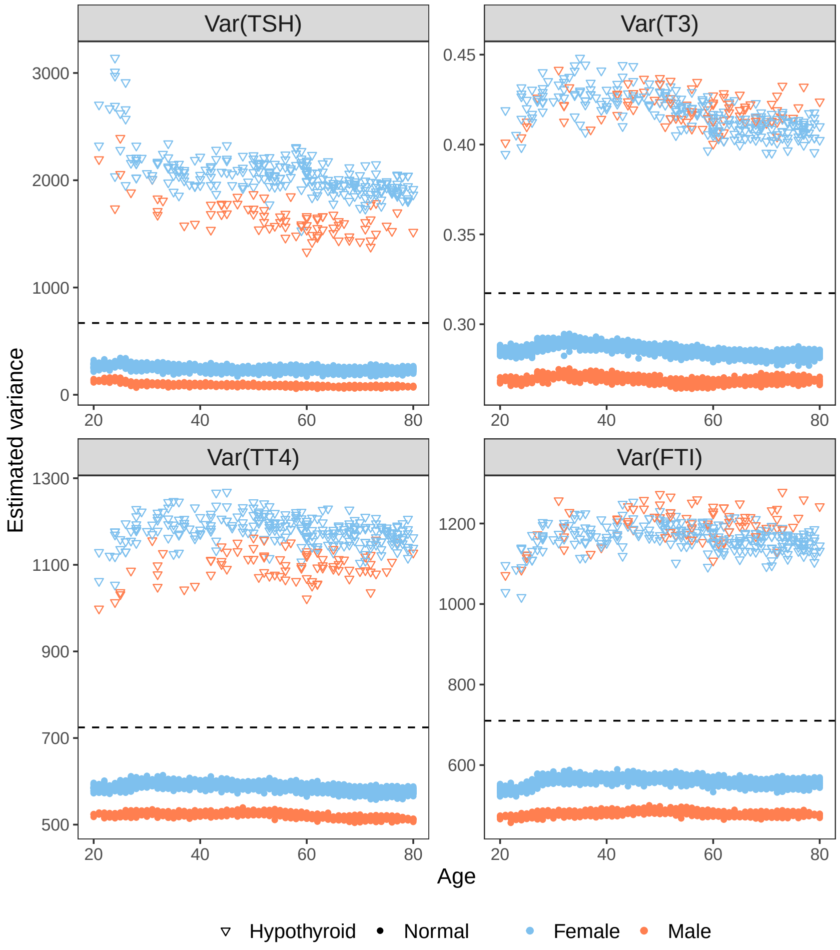

Supplementary Figure 9: Estimated variances for the four hormones as a function of age, sex and diagnosis. Dashed lines represent the sample variances computed using the whole sample.

*Corresponding author. Department of Decision Sciences, HEC Montréal, 3000 chemin de la Côte-Sainte-Catherine, Montréal (Québec), Canada, H3T 2A7. E-mail: cansu.alakus@hec.ca
