# Supplementary material for: Covariance regression with random forests
Source: BMC Bioinformatics. 2023 Jun 17;24:258. doi: 10.1186/s12859-023-05377-y (PMC10276920; doi:10.1186/s12859-023-05377-y)
Supplement: Supplementary file 8 — Additional file 8. Table presenting the computational times [file 12859_2023_5377_MOESM8_ESM.pdf]

Additional file 8 for
Covariance regression with random forests

Cansu Alakus*, Denis Larocque, Aurélie Labbe

Comparison of computational times

All simulations were run in R version 3.6.0 on a Linux machine with Intel(R) Xeon(R) E5-2667 v3 @ 3.20GHz with 396 GB of memory. The average computational time of each method for the four DGPs is presented in Supplementary Table 1. For both methods, the time for a setting consists of the time for training and the time for prediction for a new data set. We can see that the proposed method is significantly faster than `covreg`.

Supplementary Table 1: Average computational time (in seconds) of both methods over 100 replications for each simulated data set.

n_{train}	DGP	`CovRegRF`	`covreg`
50	DGP1	2.89	148.23
	DGP2	2.85	149.12
	DGP3	2.60	304.43
	DGP4	2.46	248.27
100	DGP1	4.01	151.55
	DGP2	4.01	151.97
	DGP3	3.74	283.25
	DGP4	3.44	247.77
200	DGP1	6.46	228.57
	DGP2	6.67	229.55
	DGP3	6.48	428.89
	DGP4	5.82	495.07
500	DGP1	15.07	383.16
	DGP2	14.77	384.52
	DGP3	15.38	593.00
	DGP4	13.49	744.41
1000	DGP1	52.64	771.69
	DGP2	52.34	739.52
	DGP3	62.96	984.28
	DGP4	53.95	1318.28

*Corresponding author. Department of Decision Sciences, HEC Montréal, 3000 chemin de la Côte-Sainte-Catherine, Montréal (Québec), Canada, H3T 2A7. E-mail: cansu.alakus@hec.ca
